# Stem cell dynamics and pretumor progression in the intestinal tract

**DOI:** 10.1007/s00535-016-1211-3

**Published:** 2016-04-23

**Authors:** Huiying Ma, Folkert H. M. Morsink, George Johan Arnold Offerhaus, Wendy W. J. de Leng

**Affiliations:** Department of Pathology, University Medical Center, 3508 GA Utrecht, The Netherlands

**Keywords:** Colorectal cancer, Pretumor progression, Stem cell, Dynamics, Trace

## Abstract

Colorectal carcinogenesis is a process that follows a stepwise cascade that goes from the normal to an invisible pretumor stage ultimately leading to grossly visible tumor progression. During pretumor progression, an increasing accumulation of genetic alterations occurs, by definition without visible manifestations. It is generally thought that stem cells in the crypt base are responsible for this initiation of colorectal cancer progression because they are the origin of the differentiated epithelial cells that occupy the crypt. Furthermore, they are characterized by a long life span that enables them to acquire these cumulative mutations. Recent studies visualized the dynamics of stem cells both in vitro and in vivo. Translating this work into clinical applications will contribute to the evaluation of patients’ predisposition for colorectal carcinogenesis and may help in the design of preventive measures for high-risk groups. In this review, we outline the progress made in the research into tracing stem cell dynamics. Further, we highlight the importance and potential clinical value of tracing stem cell dynamics in pretumor progression.

## Introduction

Colorectal cancer (CRC) is the third commonest cancer worldwide. In 2012, CRC was diagnosed in more than one million patients, accounting for 9.7 % of all cancers, with subsequently high global cancer mortality [1]. CRC death can be prevented by early detection of carcinomas in a curable stage, by removal of the precursor lesions, or by preventive measures in patients with well-established and well-defined risk factors. The appearance of adenomatous polyps is generally the first visible feature of CRC tumorigenesis, and removal of these polyps is in that case one of the first priorities. However, approximately 12–40 % of the adenomas appear to be flat or depressed, and they may be missed during endoscopic visualization [2]. This will hamper early detection and proper secondary prevention of CRC. Therefore, optimal measures of primary and secondary prevention require a thorough understanding of the pathogenesis, biology, and natural history of CRC.

**Fig. 1:**
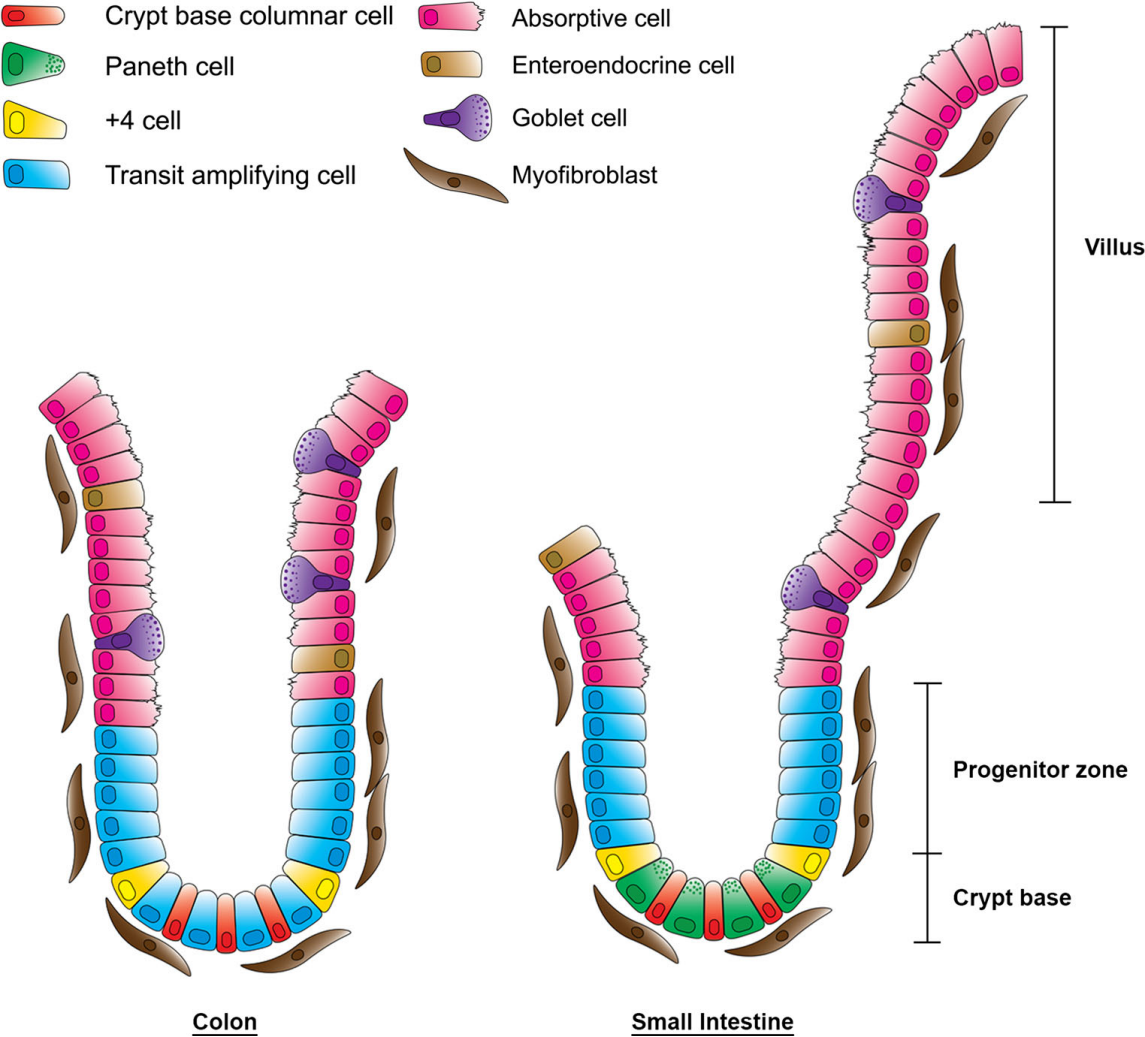
The crypt in the colon and the crypt–villus axis in the small intestine. The crypt is surrounded by a sheath of a single layer of myofibroblasts and lined with epithelial cells comprising three main types of cells: enterocyte absorptive cells, goblet cells, and enteroendocrine cells. In the small intestine, there is a fourth cell type present in the bottom of the crypt, the Paneth cell. Together with the two stem cell populations—crypt base columnar cells and +4 cells—they form the crypt base. Above the crypt base, transit-amplifying cells constitute the progenitor zone

CRCs arise in the mucosal lining of the large bowel, which consists of supportive tissue, the lamina propria, and an epithelial lining that forms multiple crypts (Fig. 1). The crypt can be considered as the smallest functional unit of the colorectal mucosa [3, 4]. The mouth or opening of the crypt is at the luminal surface of the mucosa and the base of the crypt rests on the muscularis mucosae, a tiny muscle layer that separates the mucosa from the submucosa. Epithelial cells lining the crypt are born in the basal part, or bottom, of the crypt, where cell division occurs, and which is therefore called the “proliferative compartment.” During their lifecycle, cells migrate toward the luminal surface of the crypt and they differentiate while losing their proliferative capacity. At the surface they undergo apoptosis and/or are extruded into the luminal contents of the bowel. Cross talk between the epithelial lining of the crypt and its environment occurs via the myofibroblasts that form a crypt sheath. The environment is involved in the balance between cell renewal, proliferation, migration, differentiation, and death, which occurs in a strictly regulated homeostasis along the longitudinal axis of the crypt [5].

## Adenoma–carcinoma sequence

**Fig. 2:**
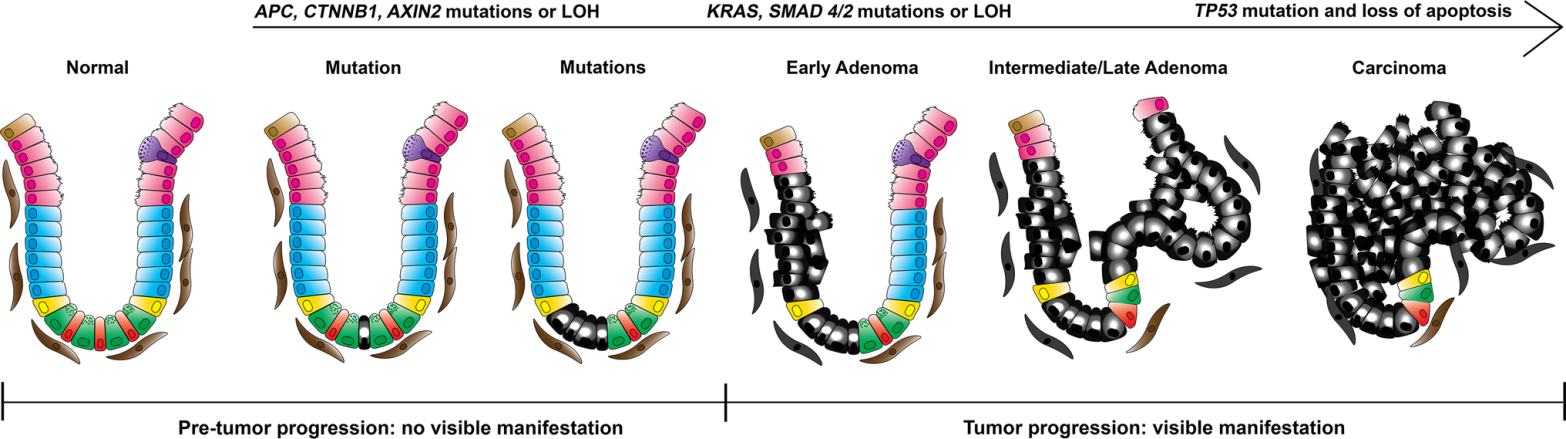
Adenoma–carcinoma sequence. Enhanced longevity of stem cells occurs in early stages of pretumor progression and it is accompanied by increasing genetic instability and accumulation of mutations. When multiple mutations are acquired, the invisible phase of pretumor progression ends and visible tumor progression begins. These preinvasive stages are grossly visible, are morphologically well defined, and can be recognized as adenomas. *LOH* loss of heterozygosity

An increasingly growing genetic instability with consecutive alterations in specific genes, such as oncogenes and tumor suppressor genes and maintenance and repair genes, leads ultimately to autonomous and invasive growth, as observed in cancer. Traditionally, colorectal carcinogenesis is described with the adenoma–carcinoma sequence; that is, a stepwise tumor progression model in which consecutive stages from normal to preinvasive stages ultimately lead to an invasive carcinoma with the capacity to metastasize because of the increasing accumulation of genetic alterations (Fig. 2). The model provides us with the information needed to investigate the timing of the genetic aberrations that accumulate and the accompanying status of cancer-related signaling pathways [6]. It has recently been made clear that adenoma formation is preceded by a time interval during which the ground (i.e., the intestinal mucosa) is fertilized for tumor growth, but without a manifestation visible to the naked eye [7]. Since the precursor lesions of the tumors are usually only visible after the age of 50 years, this implies that much of the time window suitable for preventive measures and risk assessment lies before this age.

## Pretumor progression

It thus takes a long time before a cell accumulates a sufficiently heavy mutational load to turn into a cell that is able to generate a tumor [8, 9]. Therefore, visible tumor formation is preceded by a phase called “pretumor progression” which starts from birth [10]. During a cancer patient’s life, mutations occur from birth and may remain for decades without visible changes, followed by 10–15 years of visible adenoma formation eventually progressing to colorectal carcinoma. The first mutations that occur at the very beginning of pretumor progression do not visibly alter the phenotype of the cells in the crypt. Even patients carrying germline mutations in *TP53* or *APC* at birth initially have no discernable phenotypes. Nevertheless, these two genes are among the commonest and most important tumor suppressor genes in solid tumors such as CRC [11, 12].

It is generally thought that the earliest event during pretumor progression leading to colorectal carcinogenesis occurs in the stem cell compartment. Only the stem cells can live long enough to acquire multiple mutations that are then fixed into the genome of their progeny and in this fashion are passed on to following generations. It is postulated that cancer risk directly relates to the number of stem cell divisions because the more divisions occur, the higher the chance for stem cells to gain mutations [13]. Once sufficient mutations have accumulated during the pretumor progression phase, the stem cells convert to a recognizable neoplastic cell which initiates the visible tumor progression phase. For a thorough understanding of colorectal tumorigenesis, study of stem cell behavior is a prerequisite.

## Stem cells

Stem cells are located in the stem cell niche at the bottom of the crypt and are responsible for the maintenance of crypt homeostasis by continuously replenishing the epithelial crypt lining [14] (Fig. 1). Their identity was first investigated by Cheng and Leblond [15], who called these cells, which were interspersed among Paneth cells in the small bowel, “crypt base columnar cells.” These cells are defined as a group of undifferentiated cells with the specific capacity to produce a variety of cell types, including transit-amplifying cells, which are destined to proliferate and migrate along the crypt toward the surface while differentiating at the cost of their proliferative capacity [16]. The complete life cycle of these cells takes about 5 days, and the entire epithelial lining of the gut is replaced once a week [17]. Since stem cells are the only cells capable of preserving their population as well as producing an offspring of differentiated cells that forms the epithelial lining of the intestinal crypt, their numbers must be maintained [18].

To describe how stem cells maintain their numbers—say, by homeostatic self-renewal—two different models were proposed [19, 20]. In the first model, the deterministic model, stem cells exist in the stem cell niche and each cell generates exactly one stem cell and one transit-amplifying cell by asymmetric division. Transit-amplifying cells continue to differentiate, and the stem cell in this model is “immortal,” acquiring accumulated mutations as a fast track to neoplasia, resulting in a fixed number of stem cells. A more acceptable model that was postulated recently is the stochastic model. This model proposes that each stem cell in the stem cell compartment is equally prone to become extinct over time and by chance yield zero, one, or two stem cells (corresponding to two, one, or zero transit-amplifying cells). If zero daughter stem cells are formed, the specific stem cell clone information is lost and the stem cell is replaced by the neighboring stem cell, a process which is called “neutral drift” [21, 22]. In the short term, the stem cell replacement follows this neutral drift pattern, leading to neutral competition among all stem cells instead of a hierarchical organization [23]. According to this model, new lineages appear randomly, and eventually a single ancestral stem cell lineage is maintained and occupies the entire crypt, which is called “niche succession” [24]. It is estimated that on average every 8 years niche succession will occur in the normal human colon as a result of this continuous crypt cell turnover [10, 25].

Stem cells are rapidly dividing cells living stably in the stem cell niche and continuously transferring their genetic information to the next generation, in this fashion constituting the main pool of stem cells. However, under certain circumstances, such as injury or damage, these fast-cycling stem cells are replenished by slow-cycling stem cells which will perform a similar function [26]. This alternative stem cell pool originates from cell position +4 when one counts from the bottom of the crypt and is directly located above the Paneth cell zone [27, 28]. These two subpopulations of stem cells were referred to by Cheng and Leblond [29] as the “stem cell zone model” and by Potten [27] as the “+4” model.

## Stem cell microenvironment

Stem cell behavior is also affected by the intestinal stem cell niche, which provides a microenvironment suitable for stem cells to live in. In the stem cell niche, myofibroblasts are the first layer of subepithelial cells around the crypts which can interact with the stem cells [5, 30]. By either direct contact or paracrine secretion, myofibroblasts can modulate stem cell behavior via activation of conserved signaling pathways such as the Wnt and bone morphogenetic protein pathways [5, 31–33]. Paneth cells are also found to act as key players because of their proximity to the stem cells [34, 35]. The Paneth cells produce factors such as epidermal growth factor, transforming growth factor α, and Wnt3, all essential for activation of the Wnt pathway and stem cell maintenance [36, 37]. The formation of organoids from intestinal tissue samples is markedly improved when Paneth cells are co-cultured with stem cells, and *Gfi1*^−/−^ and *Sox9*^−/−^ mice which have no apparent Paneth cells contain decreased numbers of intestinal stem cells [36, 38, 39]. However, Kim et al. [40] generated a knockout mouse model to deplete the Paneth cell component. The *Lgr5*^+^ stem cells still could continuously proliferate, differentiate, and occupy the entire bottom of the crypt without the assistance of Paneth cells. Complete loss of Paneth cells can be accomplished by inducible depletion of the transcription factor Math1, and in this situation, the maintenance and proliferation of stem cells remained normal [41]. Stem cells alternatively have an effect on Paneth cells. Depletion of *Lgr5*^+^ stem cells will result in the premature death of Paneth cells, further evidence of their close interrelationship [42]. Research on the interaction between the niche and stem cells is still in the very early stages, and more work is needed to clarify how the crypt microenvironment accommodates stem cells.

## Stem cell markers

One problem associated with tracing intestinal stem cells is that they cannot be easily identified through their morphology. Therefore, much effort has been put into identification of their specific biomarkers. Currently, it is generally accepted that leucine-rich repeat containing G-protein-coupled receptor 5 (LGR5), a G-protein-coupled receptor, is specifically expressed on the surface of crypt base columnar cells. LGR5 was found throughout the entire gastrointestinal tract [43–45]. Culturing of *Lgr5*^+^ stem cells will result in the formation of long-lived, self-organizing crypt–villus organoids [46], and *Lgr5*^+^ stem cells are the source of the continual replenishment that maintains the crypt homeostasis [47]. LGR5 also serves as an essential mediator for Wnt signal transduction by interacting with R-spondins, and in this way contributes to maintaining the stemness of stem cells [48, 49]. After ablation of *Lgr5*^+^ cells, their function may be compensated for by cells other than the *Lgr5*^+^ cells [50]. These cells turned out to be *Bmi1*^+^ cells, which are quiescently located at the +4 cell position relative to the crypt base, suggesting that *Bmi1*^+^ stem cells form a reserve stem cell pool [50]. Thus, these two distinct stem cell populations imply a model where *Lgr5*^+^ stem cells mediate homeostatic self-renewal and *Bmi1*^+^ stem cells mediate injury-induced regeneration [26, 50]. Although they are two distinct stem cell populations, there is nevertheless a bidirectional lineage relationship between active and quiescent stem cell states which implies they may mark overlapping cell populations [51–55] Furthermore, about 20 % of *Lgr5*^+^ stem cells remain quiescent and express *Lgr5* before they differentiate. If the intestine is injured, they give rise to differentiating epithelial cells; that is, the function of *Lgr5*^+^ stem cells and of *Bmi1*^+^ stem cells is not completely mutually exclusive but shows overlap [56].

**Fig. 3:**
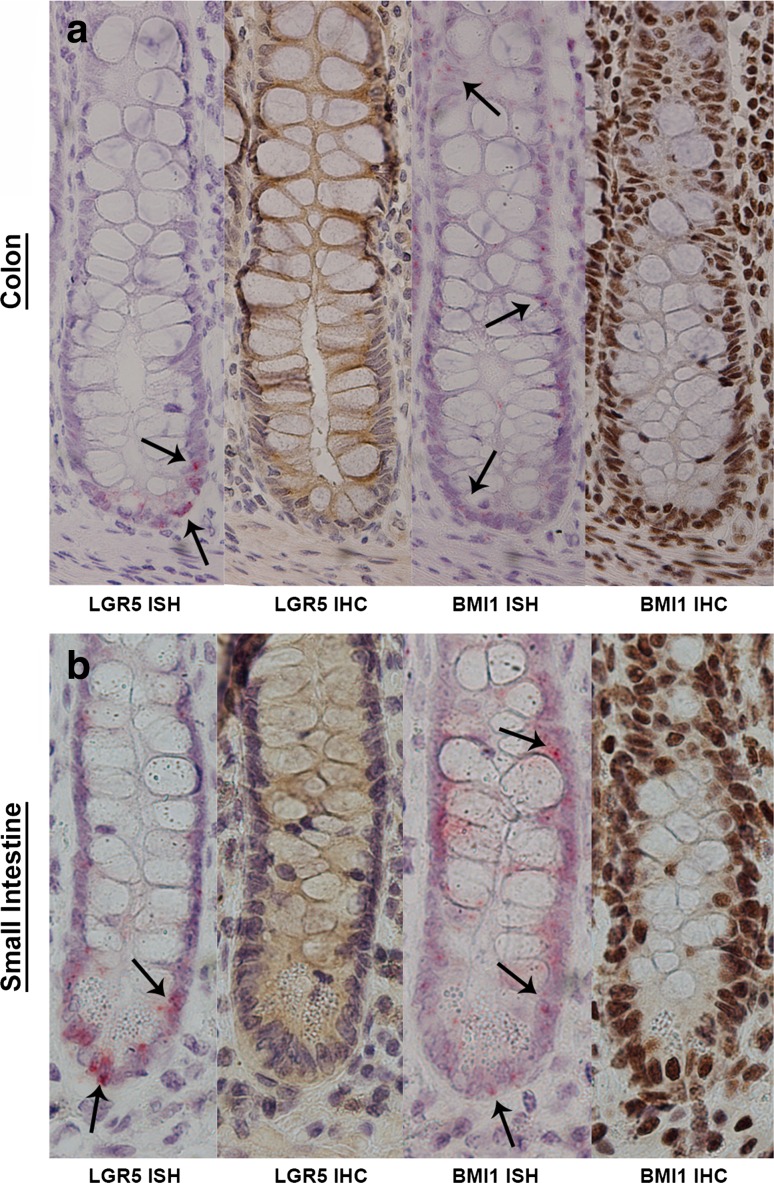
**a** Leucine-rich repeat containing G-protein-coupled receptor 5 (*LGR5*) and BMI1 staining by means of in situ hybridization (*ISH*) and immunohistochemistry (*IHC*) in the same crypt of the normal colon mucosa. **b** The same staining in the small intestine. The *red dots* indicate the location of LGR5 messenger RNA obtained with ISH. For BMI1, the ISH staining is nonspecific. Similarly, IHC gives a nonspecific staining. Magnification 200×

Expression of LGR5 in the human colorectum is extremely low, and visualization by means of immunohistochemistry is challenging, although it has been reported [57–59]; in situ hybridization of messenger RNA may therefore be a more feasible method to detect LGR5 expression [43, 60]. Here we compare the levels of LGR5 and BMI1 protein and messenger RNA expression by means of immunohistochemistry and in situ respectively (Fig. 3). It is clear that LGR5 can be detected by in situ hybridization mainly in the base of the crypt, as expected, whereas immunohistochemistry shows mostly nonspecific staining. Compared with LGR5 expression, BMI1 messenger RNA is expressed along the crypt, and is not restricted to a specific compartment or cell position. Also with immunohistochemistry, expression of BMI1 appears nonspecific. In addition to LGR5 and BMI1, more and more proteins are postulated as potential stem cell markers (Table 1).

**Table 1:** Potential stem cell markers expressed in crypt base columnar (CBC) cells and +4 cells

Marker	Reported position	Evidence
CD44	CBC cells	The *CD44* ^−/−^/*Apc* ^min/+^ mice, which lack CD44 expression, showed a significant increase in apoptotic cell numbers at the crypt base between positions 0 and +4 [61]
Msi-1 (Musashi-1)	CBC cells	Msi-1 is expressed in only a few Paneth cells of the adult mouse small intestine crypt as determined by immunohistochemistry, and the cells are also positive for Ki67 staining, which indicates their proliferative activity [62]
Olfm4 (Olfactomedin-4)	CBC cells	Olfm4 was first enriched in human colon examined by microarray analysis and then detected expressed specially in CBC cells in human small intestine and colon by means of in situ hybridization [63, 64]
ASCL2 (Achaetescute-like 2)	CBC cells	Transgenic expression of ASCL2 induces crypt hyperplasia and loss of it leads to the disappearance of *Lgr5* ^+^ SCs [65, 66]
SMOC2 (SPARC-related modular calcium binding protein-2)	CBC cells	*Smoc2* was detected in CBC cells in a *Smoc2* ^EGFP–IRES–CreERT2^ knock-in mouse model. When this mouse model was crossed with an *R26R*–*LacZ* Cre reporter mouse, the typical long-lived SCs were visualized by lineage tracing [54]
SOX9	CBC cells	*Sox9* EGFP transgenic mice reveal that *Sox9* ^EGFP^ low level expressing cells are enriched in *Lgr5* ^+^ cells. Single *Sox9* ^EGFP^ low level expressing SCs can generate organoids and continuously differentiate [67, 68]. SOX9 was also reported to limit proliferation of label-retaining cells in mouse small intestine [69]
KLF5 (Krüppel-like factors)	CBC cells	First found highly expressed in epithelial crypt cells and then recognized as a potential SC marker [70]. Depletion of KLF5 from *Lgr5* ^+^ CBC cells in adult mouse intestine leads first to halting of the proliferation of CBC cells and transit-amplifying cells, accompanied by an increase in apoptosis, and later gradual depletion of all the *Lgr5* ^+^ CBC cells [71]
LRIG1 (Leucine-rich repeats andimmunoglobulin-like domains 1)	+4 cells	Lineage tracing by intercrossing *Lrig1*-CreERT2 and *R26R*-*LacZ* mice reveals that LRIG1 marks the relatively quiescent SCs and loss of *APC* in LRIG1 cells induces multiple adenomas via regulation of ErbB signaling [72, 73]
mTert (mouse Telomerase reverse transcriptase)	+4 cells	The slowing cycling SCs in the small intestine of mTert-GFP transgenic mice are mTert positive and resistant to high-dose radiation. These mTert-expressing SCs can further give rise to *Lgr5* ^+^ SCs [22, 74]
HOPX (Homeodomain-only protein X)	+4 cells	*Hopx* knock-in mouse models were used to verify the function of mTert as a typical SC marker, and this population of SCs interconverts with *Lgr5* ^+^ SCs [52]
ID1 (Inhibitor of Differentiation 1)	+4 cells	In response to colonic injury, ID1-positive SCs hold the long-term renewal potential of the intestinal epithelium [75]
DCLK1 (Doublecortin-like kinase 1)	+4 cells	Lineage tracing experiments reveal that DCLK1 is a specific marker of tumor SCs in the polyps of *Apc* ^min/+^ mice [76]

*ASCL2* achaete–scute complex like 2, *DCLK1* doublecortin-like kinase 1, *EGFP* enhanced green fluorescent protein, *GFP* green fluorescent protein, *HPOX* homeodomain-only protein X, *ID1* inhibitor of differentiation 1, *IRES* internal ribosome entry site, *KLF5* Krüppel-like factor 5, *LRIG1* leucine-rich repeats and immunoglobulin-like domains 1, *Msi-1* Musashi 1, *Olfm4* olfactomedin 4, *mTert* mouse telomerase reverse transcriptase, *SC* stem cell, *SMOC2* SPARC-related modular calcium binding protein 2

## Stem cell dynamics

The stem cell population itself is not a static entity, and it is thought that in the intestine the different subpopulations of stem cells can replace each other, which implies an additional complexity in many dynamic biologic processes, such as inflammation, repair, and tumorigenesis. To understand the role of stem cell behavior in tumor development, the human stem cell compartment needs to be studied. Various methods have been established to describe the stem cell kinetics and dynamics in quantitative terms to provide us with tools to study their behavior, location, and numbers. Recent studies used different approaches, notably stem cell lineage tracing, methylation pattern diversity, and mitochondrial DNA mutations.

## Stem cell lineage tracing

Stem cell lineage tracing enables one to look at the progeny of a stem cell. The study of its offspring that forms a new population will uncover the pattern of the stem cell fate and record the behavior of stem cells [77]. This method can be performed by the labeling of stem cells with dyes or radioactive tracers, transfection or viral transduction of genetic markers, incorporation of stem cell markers by genetic recombination, or a recent approach that made use of multicolor reporters [78]. Whatever the technique used, the basic idea is to label the specified stem cell and trace its lineage over time. Therefore, finding the appropriate markers is the first problem that one has to overcome. They should be specific, easily detectable, and remain unaltered and stable in different microenvironments. After binding to the cells, they should not change the features of these cells, their progeny, and their neighbors. Further, they must keep their characteristics and pass them on to all progeny without transferring them to unrelated cells in their surroundings. These requirements count for the complete life cycle of the cell and its offspring [78].

The earliest studies of lineage tracing in the intestinal tract were reported by Cheng and Leblond [15] and Bjerknes and Cheng [79]. By injecting mice with ^3^H-thymidine-labeled cells, they could trace the fate of different types of cells. During cell division the labeled DNA will be incorporated and it can then be visualized by autoradiography. After 30 h, a heavily labeled columnar cell population appeared in the crypt base around cell position 5 and above, whereas after 66 h after injection, the labeled cells had migrated downward to around positions +1 to +4. From these observations it was concluded that the stem cells lie within the crypt base columnar cell population. However, this was later disputed by Potten et al. [80, 81], who found that label-retaining cells after radiation damage were positioned at the +4 cell position. They argued that these must be the stem cells because after crypt homeostasis had been established, these label-retaining cells persisted for around 4 weeks.

The identification of *Lgr5*^+^ cells as stem cells in the intestinal tract has led lineage tracing into a new era. By means of an inducible *Lgr5*^EGFP–IRES–CreERT2^ knock-in mouse model, Barker et al. [43] showed that *Lgr5*^+^ cells can give rise to all cell lineages present in the intestine and are maintained for a long time. Sangiorgi and Capecchi [82] applied the same method to find out that *Bmi1*^+^ stem cells represent a second subpopulation with a long-term self-renewal capacity that exists in the intestine. Thereafter, studies focused on the interrelationship between *Lgr5*^+^ and *Bmi1*^+^ stem cells, demonstrating that rapidly cycling *Lgr5*^+^ stem cells can be the source of slow-cycling *Bmi1*^+^ stem cells, and vice versa [50, 52], as discussed previously. By using the same approach, Schuijers et al. [83] identified a small proportion of *Lgr5*^+^ stem cells which also expressed *Olfm4*, which may be a potential marker for stem cells. Furthermore, by crossing this *Lgr5*^+^–Cre recombinase mouse model with a *Ki67*^*RFP*^ knock-in allele mouse, Basak et al. [84] showed that *Lgr5*^+^ cells are continuously in cell cycle and that the cells at the +4 position leave the cell cycle. Later Snippert et al. [47] created another exciting model to trace stem cell lineages. They labeled *Lgr5*^+^ stem cells in a mouse model with a multicolor Cre-reporter system to trace the lineages of different stem cells simultaneously in one crypt. They showed that *Lgr5*^+^ cells could give rise to all other intestinal cell lineages, and over time each crypt was occupied by only one color, implying that a single clone had eventually taken over the crypt. Also in adenomas a widespread expression of *Lgr5* was found, which suggested a potential population of stem cells [85, 86]. However, contrary to this concept, the first continuous and marker-independent clonal labeling system identified fewer functional stem cells in the normal murine intestine as well as in adenomas, consistent with the notion that only a small amount of stem cells participate in tumor formation [87]. The above-mentioned studies were performed in mice, and the method can of course not be applied to human tissues. Although there are many similarities between mice and humans, there are differences too [88]. Therefore, other methods were developed to study stem cell lineages in humans.

## Methylation pattern diversity

One approach for visualizing stem cell dynamics in humans is to analyze the diversity of methylation patterns. Methylation is commonly studied for its influence on gene expression. However, methylation events also occur at CpG sites in inactive genes in the tissue of interest. Since the gene is inactive, methylation is less tightly regulated and occurs randomly during the replication of DNA strands in the stem cells, providing an epigenetic signature to the stem cell lineage. The diversity in the location and the number of methylated CpGs in methylation tags in a nonfunctional gene and the number of methylated CpGs will increase with the longevity of the stem cell lineage and the number of stem cell divisions [89]. Thus, the history of a crypt can be recorded by the study of these methylation patterns as epigenetic signatures [90–92]. Each crypt contains various stem cell lineages which are constantly changing in a dynamic way [58]. This method of investigating stem cell lineages by means of the methylation diversity has been described by Yatabe et al. [93] and Kim et al. [24].

The longer a stem cell lineage has resided in the niche, the greater the chance that (epi)genetic changes occur and therefore the greater the diversity of methylation patterns that exist. For example, a greater diversity of methylation patterns was shown in the nonexpressed *NKX2-5* gene (also known as *CSX*) in the crypts of normal-appearing mucosa of familial adenomatous polyposis (FAP) patients, who have a germline mutation in the *APC* gene that inhibits Wnt activation, compared with colons of normal controls, indicating that stem cells were present for a prolonged period in FAP patients during which they acquired these methylation events. This increase in methylation diversity is therefore indicative of the fact that niche succession occurs less frequently in FAP patients than in controls [94]. A mathematical model showed that the estimated time between niche succession is 32 years in FAP crypts but 8 years in normal colon crypts [10]. This extended longevity of the stem cell lineages in FAP patients explains the higher risk of colon cancer, indicating that methylation diversity can be used as an epigenetic molecular clock to record the history of stem cells. Increased longevity of stem cell lineages and increased number of stem cell divisions are therefore associated with a higher risk of the accumulation of mutations and initiation of colorectal carcinogenesis. This method of studying stem cell dynamics by means of determination of methylation pattern diversity is a valuable tool in research and could eventually be useful in a diagnostic setting to predict the risk of tumor formation. For now, it is not yet feasible to apply this technique in routine diagnostics because the entire procedure is time-consuming and tedious.

## Mitochondrial genome

An alternative way of visualizing stem cell dynamics in the intestine is the study of the mutation rate in the mitochondrial genome. Unlike other organelles, mitochondria contain multiple copies of their own circular genome, mitochondrial DNA, in the mitochondrial matrix [95]. Induced by environmental DNA-damaging agents, such as free radicals from the respiratory chain, endogenous mitochondrial reactive oxygen species, and certain drugs, somatic mitochondrial DNA mutations occur as a general phenomenon and are easily accumulated because of the lack of histone protection and limited DNA repair capabilities [96]. This accumulation is random, and increases with age [95, 97]. Therefore, the number of mutations in the mitochondrial genome can be used as a biomarker to study the dynamics of stem cells. Besides sequencing of the mitochondrial genome, stem cell lineages with mitochondrial DNA mutations can be recognized by visualization of mitochondrial enzyme activity. The largest mitochondrial gene, that encoding cytochrome *c* oxidase (COX), is most prone to be inactivated by a mutation. Inactivation of the enzymatic activity of COX can be visualized by dual-color COX and succinate dehydrogenase enzyme histochemistry [95]. In this method, enzyme histochemistry is simultaneously applied for COX (brown) and succinate dehydrogenase (blue), another enzyme of the respiratory tract. Cells mutated for COX appear blue because of the lack of brown COX staining, whereas COX wild type cells will appear brown. Using this method, Gutierrez-Gonzalez et al. [98] identified some partially mutated small intestinal crypts, providing evidence that these crypts contain multiple stem cell lineages [99]. COX-mutated crypts were further found in clusters throughout the entire colon, where the size of these clusters, which are called “patches,” increased with age [100]. Thus, stem cell dynamics can be assessed in situ with simple enzyme histochemistry.

## Stem cell dynamics in pretumor progression

Since stem cells serve as the primary source to carry and pass on mutations leading to intestinal tumor formation, visualizing stem cell dynamics is an effective way to study and monitor tumorigenesis. Understanding the role of stem cells in pretumor progression might provide us with an effective way to investigate and predict the natural history and risk of tumor occurrence. Hereditary CRC syndromes with a well-established risk of developing CRC are suitable human disease models for application of our research tools to look at pretumor progression and stem cell dynamics in comparison with normal controls.

Inherited intestinal tumor syndromes, including FAP, Peutz-Jeghers syndrome (PJS), juvenile polyposis syndrome, Lynch syndrome, and sessile serrated polyposis contribute between 2 and 5 % of CRC cases [101]. Patients with these inherited syndromes carry the first genetic alteration with the accompanying risk from birth, and these syndromes can therefore be considered as relatively well-defined pretumor progression models. Germline mutations leading to hereditary CRC will affect stem cell behavior and cause an accelerated pretumor progression phase. Indeed, in FAP and PJS, the longevity of the stem cells, visualized by study of the diversity of methylation patterns, appeared considerably increased in the normal-looking intestinal mucosa, compared with healthy controls [102]. Increased longevity is accompanied by an increased predisposition for accumulated additional mutations and subsequent tumor progression [94].

### Stem cells in FAP models

FAP is a syndrome caused by a germline mutation of the “gatekeeper” tumor suppressor gene *APC* (which encodes adenomatous polyposis coli), where one inherited defective *APC* allele leads to progressively growing intestinal neoplasia [45]. In its classic form it is characterized by numerous adenomatous polyps in the colorectum and individuals with FAP have a virtually 100 % lifetime risk of developing CRC when no prophylactic surgical removal of the large bowel is performed [103, 104]. Haploinsufficiency of *APC* due to a germline mutation in FAP is associated with crypts that display increased crypt fission and an increased number of stem cells [3, 105]. Baker et al. [106] showed that the number of stem cells further increased in *APC*^−/−^ crypts compared with *APC*^+/−^ crypts. Furthermore, the loss and replacement rate of the *APC*^−/−^ stem cells is enhanced, and this accelerated division rate ultimately results in the accumulation of mutations leading to the cancer-prone state. That haploinsufficiency of *APC* leads to the above-mentioned manifestations may be explained by the observation that *APC* influences the mitotic spindle orientation and thereby the balance between asymmetric and symmetric stem cell divisions [107–109].

APC also acts as a key factor in the Wnt signaling pathway, essential to maintain the physiological homeostasis of the stem cell niche [110]. In a complex with axin and glycogen synthase kinase 3β, APC forms the key destruction complex of the Wnt signaling pathway through phosphorylation and subsequent degradation of β-catenin [111]. Mutational inactivation of *APC* results in the accumulation of β-catenin in the cytoplasm and translocation to the nucleus, where it forms a complex with TCF1 that acts as a transcription factor that activates the Wnt target genes ultimately leading to tumorigenesis [112]. APC is therefore an inhibitor of Wnt signaling and *APC* mutations lead to aberrant Wnt activation and stimulation of stemness in the stem cell niche [113, 114]. The self-renewal capacity of embryonic stem cells can be enhanced by modulation of APC dose-dependent Wnt signaling [115]. Dow et al. [116] found that restoration of APC-regulated normal Wnt signaling can cause tumor cells to revert to functional normal cells. These facts lend support to the use of FAP as a model for studying stem cells during pretumor progression.

### Stem cells in PJS models

PJS is another inherited polyposis syndrome, caused by a germline mutation in the *STK11* gene (also known as *LKB1*), with an increased cancer risk, both intraintestinal and extraintestinal, and it is typically accompanied by mucocutaneous skin pigmentations [117, 118]. A mutated *STK11* gene leads to loss of polarity of differentiated epithelial cells [119–121] and a deficiency in p53-mediated intestinal epithelial cell apoptosis [122]. Mouse models carrying one mutated *STK11* allele are prone to polyp and tumor formation [123, 124]. When stem cell dynamics in PJS patients compared with normal controls were analyzed with use of a methylation assay of the inactive *NKX2-5* gene in the intestinal mucosa, an increased methylation diversity was found. This indicates that niche succession is prolonged and mutations can more easily accumulate in the stem cells of PJS patients [102]. The progenitor zone was also expanded, consistent with an altered balance between division and differentiation in the epithelial lining of the intestine in PJS patients. This misbalance between cell division and differentiation may result in polyp and tumor growth [125]. The precise mechanism of tumorigenesis due to deficiency of serine/threonine kinase 11 (encoded by *STK11*) is still puzzling, but it appears that there is a link between serine/threonine kinase 11 and stem cell behavior in the gut. Similarly, a link seems to exist between serine/threonine kinase 11 and the hematopoietic stem cell population [126, 127].

## Conclusion

Identification of pretumor progression by means of visualization of stem cell dynamics seems to be an effective way to assess cancer risk. However, the invisible phenotype of pretumor progression makes this challenging. On the basis of the adenoma-carcinoma sequence, accumulating mutations during pretumor progression contribute to tumor formation. These mutations are acquired and expanded in cells that need to live long enough to build up a mutational burden. This makes stem cells an attractive study object since they are considered the primary population where tumorigenesis is initiated. Because of the important role stem cells play in pretumor and tumor progression, it is essential for researchers to focus on tracing the dynamics of these stem cells. Although there are several techniques to visualize this, as summarized in this review, they all provide indirect evidence, are labor-intensive and tedious, and are therefore mostly applied in model organisms [128, 129]. Some approaches allow studies in the human intestinal mucosa, and these will, in conjunction with the animal and in vitro studies, ultimately increase our understanding of the stem cell dynamics in pretumor progression and provide valuable information for risk assessment and prevention of intestinal tumorigenesis.
